# Marine Predator Algorithm-Based Optimal PI Controllers for LVRT Capability Enhancement of Grid-Connected PV Systems

**DOI:** 10.3390/biomimetics9020066

**Published:** 2024-01-23

**Authors:** Hazem Hassan Ellithy, Hany M. Hasanien, Mohammed Alharbi, Mohamed A. Sobhy, Adel M. Taha, Mahmoud A. Attia

**Affiliations:** 1 Electrical Power and Machines Department, Faculty of Engineering, Ain Shams University, Cairo 11517, Egypt; hazem.ellithy@eng.asu.edu.eg (H.H.E.); mohamed_sobhy@eng.asu.edu.eg (M.A.S.); adel-taha@eng.asu.edu.eg (A.M.T.); mahmoud.abdullah@eng.asu.edu.eg (M.A.A.); 2 Faculty of Engineering and Technology, Future University in Egypt, Cairo 11835, Egypt; 3 Electrical Engineering Department, College of Engineering, King Saud University, Riyadh 11421, Saudi Arabia; mohalharbi@ksu.edu.sa

**Keywords:** photovoltaic, low-voltage ride-through, Marine Predator Algorithm

## Abstract

Photovoltaic (PV) systems are becoming essential to our energy landscape as renewable energy sources become more widely integrated into power networks. Preserving grid stability, especially during voltage sags, is one of the significant difficulties confronting the implementation of these technologies. This attribute is referred to as low-voltage ride-through (LVRT). To overcome this issue, adopting a Proportional-Integral (PI) controller, a control system standard, is proving to be an efficient solution. This paper provides a unique algorithm-based approach of the Marine Predator Algorithm (MPA) for optimized tuning of the used PI controller, mainly focusing on inverter control, to improve the LVRT of the grid, leading to improvements in the overshoot, undershoot, settling time, and steady-state response of the system. The fitness function is optimized using the MPA to determine the settings of the PI controller. This process helps to optimally design the controllers optimally, thus improving the inverter control and performance and enhancing the system’s LVRT capability. The methodology is tested in case of a 3L-G fault. To test its validity, the proposed approach is compared with rival standard optimization-based PI controllers, namely Grey Wolf Optimization and Particle Swarm Optimization. The comparison shows that the used algorithm provides better results with a higher convergence rate with overshoot ranging from 14% to 40% less in the case of DC-Link Voltage and active power and also settling times in the case of MPA being less than PSO and GWO by 0.76 to 0.95 s.

## 1. Introduction

### 1.1. General Overview of PV Grid-Connected Systems

The shift to sustainable energy production has resulted in a massive rise in the electricity grid’s volume of renewable energy sources. These ecologically friendly energy sources help minimize carbon emissions and fulfill the ever-increasing need for electricity [1,2,3]. Photovoltaic (PV) systems have become a cornerstone in the shift to renewable energy sources as solar power technology, providing multiple advantages to the grid, the environment, and society [4]. Despite pandemic repercussions and a spike in global commodity prices that disrupted renewable energy supply chains, renewables recorded another year of record capacity expansion, although some initiatives were pushed back. As energy costs rose rapidly in late 2021 and during the Russia–Ukraine conflict in early 2022, the role of renewables in increasing energy security and sovereignty by replacing fossil fuels became key to talks [5].

For the fourth consecutive year, investment in renewable energy and fuels increased to USD 366 billion, and a record growth in global electricity output resulted in the delivery of more than 10% of the world’s electricity through wind and solar power [6]. Amid supply challenges, shipping holdups, and soaring costs for solar and wind energy parts, the expansion of renewable energy capacity increased by 17% in 2021 to a record high exceeding 314 gigawatts (GW). The total installed capacity from renewable sources increased by 11%, reaching roughly 3146 GW, but this is still far short of the deployment required to keep the world on pace to achieve net zero emissions by 2050. Renewables produced 28.3% of global power in 2021, the same as in 2020 (28.5%) and an increase from 20.4% in 2011 [6]. Solar PV continued its record-breaking trend, adding a further capacity of 175 GW in 2021, bringing the total to roughly 942 GW [7]. Researchers have been intrigued by the grid-connected PV system because it provides sustainable and clean energy, particularly in remote regions. This system has numerous benefits, including the precision and dependability with which it generates power. Since grid-connected PV systems can cover the energy demands of multiple industries, they are becoming increasingly popular as power demand grows [8,9,10].

### 1.2. Incitement and Motivation

However, one of the major challenges of integrating PV systems with the grid is maintaining grid stability [11], particularly during voltage sag periods. This ability is known as low-voltage ride-through (LVRT) [12]. Regulatory developments related to LVRT in PV systems are assessed during the initial phase. Papers such as Sánchez and Gómez (2014) [13] and Teodorescu et al. (2010) [14] discuss the importance of grid codes to define the behavior of power plants during grid faults. In some regions, recent grid codes require PV systems to have LVRT capabilities similar to conventional power plants [15], which poses a significant challenge to the PV industry [16]. Maintaining output during these periods can be challenging for renewable energy sources, especially wind and photovoltaic systems [17], due to their inherent intermittency and sensitivity to grid conditions. Therefore, enhancing the LVRT capability of these systems through the development of control strategies is a key focus of modern power system research [18]. Various control strategies have the potential to be employed to improve the LVRT ability of the grid [19]. These strategies typically focus on managing the operation of power electronic devices, like inverters [20], which interface renewable energy systems with the grid. One of these strategies is fault ride-through control. This control technique involves a quick disconnection and reconnection of the renewable energy system during a fault [21], allowing the system to ride through the low-voltage period without tripping off. While this method can help maintain system stability, it requires precise control and robust protective mechanisms to prevent damage to the system during disconnections. Another strategy that is widely used for LVRT enhancement is reactive power control [22]. By injecting reactive power into the grid during voltage sags, this method can support grid voltage and enhance the system’s reliability by providing the grid with reactive power during voltage sags [23]. However, this approach requires careful coordination and control to prevent overcompensation, which could result in a voltage rise instead [24]. ESS (Energy Storage Systems) [25], namely batteries or supercapacitors, can be implemented to maintain power output during voltage sags, effectively enhancing LVRT capability [26]. The control of these systems involves managing the charge and discharge processes according to grid conditions. While effective, energy storage systems can be costly, and their lifetime can be a concern [27]. Another common method is active power curtailment. This is done during a fault by trimming the active power fed to the grid [28]. This reduction eases the stress on the system, allowing it to recover without reaching critical voltage levels. After the fault is resolved, the system can gradually restore the power output. This strategy is most effective when combined with other control measures, as it alone may not be sufficient to handle severe faults. Proportional-integral (PI) controllers, a staple in control systems, are proving to be an effective solution [29]. The inverter, which converts the direct current produced by the PV cells into an alternating current suitable for grid integration [30], is considered an essential component in PV systems. The inverter’s ability to manage fluctuations and disturbances in the grid is critical for LVRT capabilities. This is where PI controllers come into play. PI controllers are commonly employed in feedback control systems [31]. They adjust the system’s output based on the discrepancy between the desired and actual outcome, known as the error signal [32]. This is done in two ways: the proportional part responds to present errors, while the integral part anticipates future errors based on past errors. Combining these two actions provides a robust control mechanism that enables swift and effective responses to grid disturbances. PI controllers [33] facilitate the LVRT ability of these systems in several ways due to their simple structure, limited cost, and smooth implementation [34,35]. First, by controlling the outcomes of the inverter, namely voltage and current, PI controllers can help to maintain power quality during fault conditions. They also enhance the stability of the grid. This can prevent the system from tripping and disconnecting from the grid during a voltage dip, thus enhancing its LVRT capability. Second, by properly tuning the gains of the PI controller, both proportional and integral, the system can quickly respond to voltage dips and recover once the fault is cleared. This can minimize the duration and impact of voltage dips on the grid, further enhancing the system’s LVRT capability [36]. This paper introduces an authentic MPA application to improve the LVRT behavior of on-grid PV systems. The suggested system employs a DC–DC boost converter and a fractional open-circuit voltage technique based on a PI controller to track the maximum power point. In conjunction with a vector-control approach, the PV system grid-side inverter manages the DC-link voltage and the point of common coupling (PCC) voltage. Also, the control strategy involves using an overvoltage protection scheme to limit the overvoltage happening at the fault clearance time. The ideal set of PI controller settings is obtained. The PI unknown gains (proportional and integral) are optimized using the proposed MPA algorithm utilizing MATLAB software. The proposed control plan is validated first by running it on the system, and then by exposing the system to a 3LG (three-line to ground) fault. The usefulness of the optimum control strategy is demonstrated by comparing simulation outcomes to the outcomes obtained by the PSO and GWO algorithms.

### 1.3. Literature Review and Research Gaps

In research [37,38], the PI controller settings are determined using the technique of trial and error. This depends on the competence and understanding of the designer. Standard tuning methods, including Ziegler–Nichols, Cohen–Coon, and self-tuning fuzzy PI-based M-constrained integral gain optimization, can be used to find the best values for PI controller parameters. However, the robustness of the controller is reduced, leading to inaccurate results because of the insufficient process information dealing with mathematical models and trial and error. Due to this unconventional precision for designing a controller, meta-heuristic algorithms are an excellent alternative for dealing with this design issue [39]. This is to improve the grid-connected PV system LVRT ability. This capability includes criteria for a transient response. This criterion includes the voltage response’s settling time, percentage overshoots, undershoots, and steady-state error. Meta-heuristic algorithms were used in multiple power-system applications. These algorithms include the Genetic Algorithm (GA), which is used in optimal power flow in [40], particle swarm optimization (PSO), which is used in economic power dispatch, reactive power optimization, optimal power flow, and other power-system applications [41], the technique of bacterial foraging (BF) used in power-distribution restoration [42], artificial bee colony (ABC) employed in optimal power sharing in microgrids [43], the whale optimization algorithm (WOA) deployed in single- and multi-objective optimal power-flow issues [44], the shuffled frog leaping algorithm (SFLA) used in the economic load dispatch optimization problem [45], the hybrid technique of firefly and pattern search employed for Static Synchronous Series Compensator (SSSC)-based power oscillation damping controller design [46], the harmony search algorithm (HSA) used in the optimal placement for Flexible AC Transmission Systems (FACTS) devices to improve power systems [47], the artificial fish swarm algorithm (AFSA) in the power-system state estimation [48], the trust region reflective (TRR) algorithm used for sizing a microgird with storage [49], and the Cuckoo Search (CS) algorithm employed in the Optimal Placement and Sizing of Static Var Compensator in Large-Scale Power Systems [50]. All of these techniques aim to minimize errors. But each one of them has its own merits and limitations. The finite length of the bit string makes finding actual values for the variables complex in the standard GA despite the simplicity benefit. The main disadvantage of traditional differential evolution (DE) is the premature parameters’ convergence to local minima. This limitation can be overcome through the introduction of a penalty function. The starting population significantly influences HSA; the PSO’s lack of a proper mechanism for balancing local and global particle searches makes it prone to early convergence, and ABC reacts poorly under operational conditions. Since it does not employ local search to improve the pace of convergence when the limited search is near local or global minima, CS has a sluggish rate of convergence. It identifies authentic parameter solutions through the exclusive dependence on Lévy flight. It was determined for AFSA that the aggregation of some local optima fish resulted in a delayed convergence speed. The PI controller parameters are determined by trial and error in studies [37,38], which mainly depends on the designer’s competence and understanding. Since this is not the conventional precision for designing a controller, meta-heuristic algorithms, either evolutionary or swarm intelligence approaches, are an excellent alternative for dealing with such design issues.

Recently, novel techniques were added. These techniques include the liver cancer algorithm (LCA) [51] and the slime mould algorithm (SMA) [52]. The SMA algorithm was invented due to the fluctuating behavior of slime mould in nature. It provides an optimal pathway for connecting food with high exploration and exploitation ability. The moth search algorithm (MSA) [53], inspired by the phototaxis and Lévy flights of the moths, is also a new metaheuristic algorithm. A general-purpose population-based optimization technique called Hunger Games Search (HGS) is proposed in [54] with a simple structure, special stability features, and very competitive performance to realize the solutions to constrained and unconstrained problems effectively. The proposed HGS is designed according to the animals’ hunger-driven activities and behavioral choices. The Runge–Kutta method (RUN) was introduced in [55]. The colony predation algorithm (CPA) [56] utilizes a mathematical mapping following the strategies used by animal hunting groups, such as dispersing prey, encircling prey, supporting the most likely successful hunter, and seeking another target. The weighted mean of vectors was introduced in [57]. Harris–Hawks optimization (HHO) was presented with several applications in [58]. To fulfill the LVRT need for power balancing during various grid-failure conditions [59,60], a probabilistic wavelet fuzzy neural network (PWFNN) and a Takagi–Sugeno–Kang probabilistic fuzzy neural network (TSKPFNN) is presented. Discrete Fourier series-based control was also introduced in [61]. This study’s modeling and experimental findings proved that this technique can stabilize voltage under grid disruption. The disadvantage of this strategy is the complexity of the controller in dealing with the uncertainties of the PV system. Meanwhile, work on neuro-fuzzy controllers to improve LVRT has been described in [62]. The suggested controller is found to be successful in generating the requisite reactive power. However, the output current at the PCC side is absent. An ingenious technique, the Marine Predator Algorithm [63], was introduced to overcome the shortcomings. The extensive foraging strategy of ocean predators is the primary driver for MPA, namely Lévy and Brownian movements, and the optimum strategy for the rate of encounter in biological interactions involving prey and predator. MPA adheres to the criteria that typically regulate an optimized strategy for foraging and encounter rate policy in marine habitats. The salient feature of the MPA is its high convergence capability in reaching the global minimum point from the first phase, where it relies on its high level of exploration and exploitation capabilities [64]. MPA’s performance was assessed on 29 test functions [65], the CEC-BC-2017 test suite, a landscape generated at random, three criteria of engineering, and two challenges of practical engineering design for ventilation and energy efficiency in buildings.

### 1.4. Contribution and Paper Organization

This article contributes to having a novel approach to enhance the LVRT capability by improving the performance of the grid-side inverter while also strengthening the DC–DC boost converter behavior while maintaining an improved range of overshoot, settling time, and steady-state error.

The paper is organized in the following way: Section 2 announces the on-grid model of the PV system. It demonstrates the components and control strategy of the system. Section 3 explains the issue and illustrates the MPA technique. Section 4 announces the outcome of the simulation, while Section 5 states the comparison between the method used and other techniques used to verify the results. In Section 6, the conclusion is discussed.

## 2. Materials and Methods

### 2.1. System Modelling

**Figure 1:**
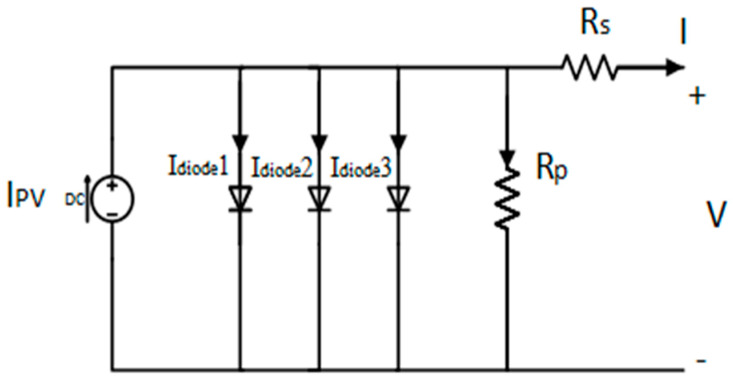
TD PV Model.

Since PV arrays are built from PV solar cells in shunt or series configurations, they may be interpreted as an electric circuit [66]. The triple diode (TD) PV cell model is used as it has high accuracy [67]. In this module, three diodes are used, with the first of them representing the losses in the emitter and bulk in the P–N junction due to recombination and diffusion, the second representing the effect of charge recombination, and the third representing the grain boundaries and defect region’s impact [68]. Meanwhile, the resistance depicts other kinds of losses [69]. The triple diode model is represented in Figure 1, while the Model I–V characteristics are illustrated utilizing the below equation:

(1) $$I=I_{\mathrm{pv}}\;–\;Io_{1}\;\{e^{\left[\frac{\left(V+IR_{s}\right)}{a_{1}V_{th}}\right]}-1\}\;–\;Io_{2}\;\{e^{\left[\frac{\left(V+IR_{s}\right)}{a_{2}V_{th}}\right]}-1\}\;–\;Io_{3}\;\{e^{\left[\frac{\left(V+IR_{s}\right)}{a_{3}V_{th}}\right]}-1\}-\frac{\left(V+IR_{s}\right)}{R_{p}}$$

where *I_pv_* represents the photocurrent of the cell, Io_1_, Io_2_, and Io_3_ represent the reverse saturation currents, while a_1_, a_2_, and a_3_ depict the three diodes ideality factors, and V_th_ = N_s_ kT/q depicts the thermal voltage. N_s_ is the number of series-connected cells in the module, k is the Boltzmann constant, and q is the electron’s charge.

**Figure 2:**
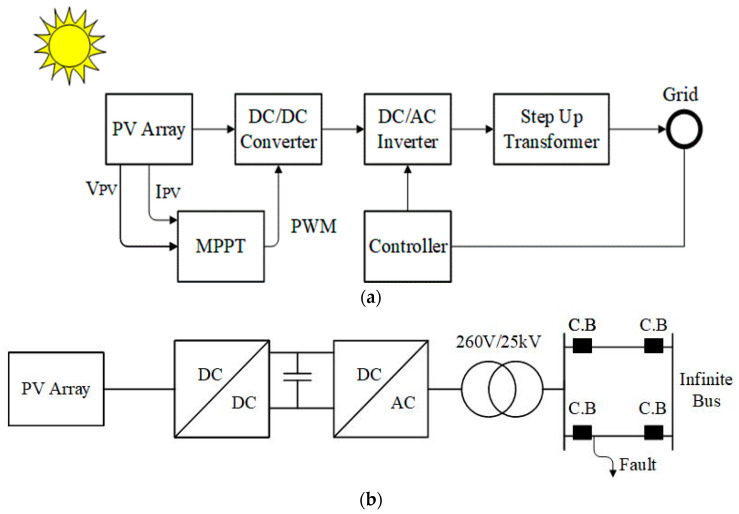
100 KW grid-connected PV System. (**a**) General, (**b**) system under study.

The mathematical analysis of the TD model is explained in [70] in nominal and real-life circumstances. A 100 kW is constructed from a Kyocera KC200GT PV module. In Table 1, the module’s electrical attributes under STCs are stated. MATLAB environment manages the whole system, with its components being the PV array, DC link capacitor, DC–DC Boost Converter, grid-side inverter, and step-up transformer. Then, a double transmission line connects the system to the grid shown in Figure 2a,b. Further information on the system is provided in Table 2.

### 2.2. Control Strategy of the System

#### 2.2.1. DC–DC Boost Converter

**Figure 3:**
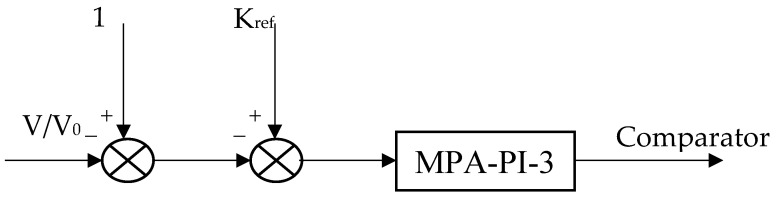
DC–DC Boost Converter.

The suggested proportional-integral (PI) controller controls the boost DC–DC converter duty cycle in the described system to achieve MPPT (maximum power point tracking) using the Incremental Conductance-Integral Regulator technique [71], which is a clear and precise method. Figure 3 depicts the control strategy introduced utilizing the MPA-based PI controller. A modulating carrier triangle waveform with a frequency of 4 kHz is used to compare the controller’s output signal. This forges the IGBT switch-firing pulses.

The following equation determines the reference duty cycle [72]:

(2) $$K_{ref}=1-\frac{N_{M}K_{M}V_{OC\text{-}pilot}}{V_{o\text{-}conv}}\;$$

where K_M_ denotes a proportionality constant between 0.71 and 0.78, as suggested in [72], V_OC-pilot_ denotes the pilot module open circuit voltage, and V_o-conv_ stands for the DC converter output voltage.

#### 2.2.2. Overvoltage Protection

**Figure 4:**
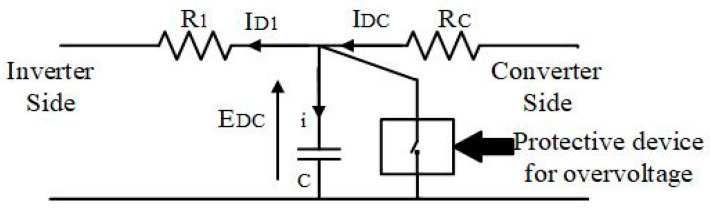
Configuration of DC link overvoltage protection.

A protective circuit is connected between the capacitor terminals [73]. This structure is shown in Figure 4. In the event that EDC surpasses the set higher limit, EDC_MAX, the resistor load (Rsh) short-circuits both capacitor terminals, absorbing energy and suppressing the voltage rise. Table 3 shows the excitation circuit ratings used in the simulation, including these protection circuit setting parameters.

#### 2.2.3. Grid-Side Inverter

**Figure 5:**
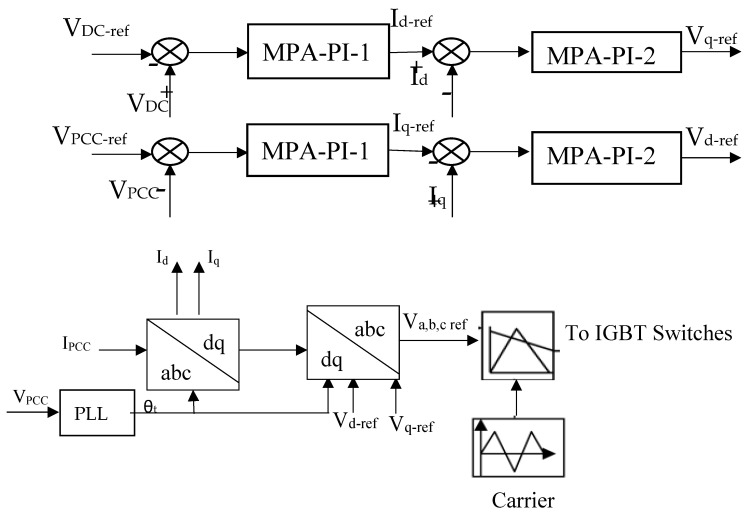
Voltage grid-side inverter cascaded control.

The switching from AC to DC signals is accomplished through a two-level, three-phase system featuring six IGBT switches [74]. The suggested MPA-based PI controller is coupled with a control method formed of two cascaded loops during the process. The cascaded control system is distinguished by its simpler architecture. PI controllers for every loop may be developed individually, and the system’s nonlinearity and parameter fluctuations can be handled efficiently [75]. The control approach is entirely conducted in the d-q rotating frame. A phase-locked loop is employed to calculate the rotation frequency, which is then accommodated to yield the angle of rotation required to switch the three-phase current and voltage to the d-q frame. The outer loops of this control strategy regulate the terminal voltage at PCC (VPCC) and voltage of the DC-link, which remain constant at 500 V throughout the operating process. As seen in Figure 5, the inside loops regulate the d-q inverter currents. The suggested MPA-based PI controllers (PI1 and PI2) are employed to attain control aims.

## 3. Used Optimization Algorithms

### 3.1. Marine Predator Algorithm

#### 3.1.1. MPA Interpretation

MPA, like most metaheuristics, is a population-based approach. The initial solution is equally dispersed across the search space in an equal fashion as the first trial:

X_0_ = X_min_ + rand (X_max_ − X_min_) (3)

X_max_ and X_min_ depict the higher and lower bounds of the variable, and rand stands for a random uniform vector with a range between 0 and 1.

Following the survival of the fittest argument, apex predators are superior at foraging in nature. A top predator is nominated as the best solution to design the matrix of Elite. The arrays of this matrix depend on data about the prey’s location to locate prey.

(4) $$\mathrm{Elite}={\left[\begin{array}{cccc} X_{1.1}^{1} & X_{1.2}^{1} & \ldots & X_{1.d}^{1}\\ X_{2.1}^{2} & X_{2.2}^{2} & \ldots & X_{2.d}^{2}\\ \ldots & \ldots & \ldots & \ldots \\ \ldots & \ldots & \ldots & \ldots \\ X_{n.1}^{1} & X_{n.2}^{1} & \ldots & X_{n.d}^{1} \end{array}\right]}_{nxd}$$

where $\vec{X_{1}}$ is the most significant predator vector, which is copied n times to construct the matrix of Elite. Further, n is the search agent’s number, while the number of dimensions is d. Predators and prey are regarded as search agents due to the prey hunting for its meal while searching for a predator for the prey. The Elite will be upgraded if a better predator replaces the top predator after each cycle.

Another matrix with the exact dimensions as Elite is Prey. Predators use it to keep track of their locations. In a nutshell, the initialization generates the initial prey, and then the Elite is built by the fittest predator. The prey is displayed below:

(5) $$Prey={\left[\begin{array}{cccc} X_{1.1} & X_{1.2} & \ldots & X_{1.d}\\ X_{2.1} & X_{2.2} & \ldots & X_{2.d}\\ \ldots & \ldots & \ldots & \ldots \\ \ldots & \ldots & \ldots & \ldots \\ X_{n.1} & X_{n.2} & \ldots & X_{n.d} \end{array}\right]}_{nxd}\;$$

**Figure 6:**
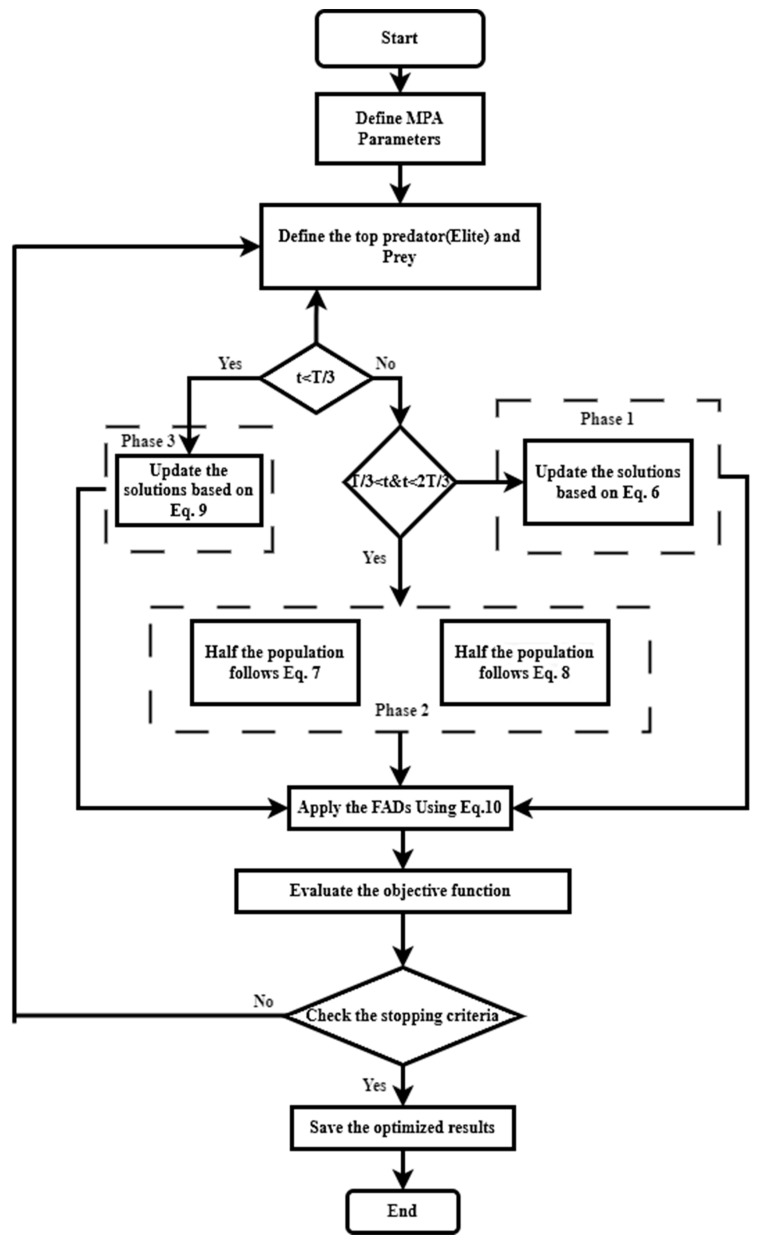
Flowchart of MPA (Marine Predator Algorithm).

X_i,j_ in Equation (5) stands for the j_th_ dimension of the i_th_ prey. It should be emphasized that the entire optimization process is primarily straight tied to both matrices. A flowchart of the algorithm is displayed in Figure 6.

#### 3.1.2. MPA Optimization Scenarios

Three primary optimization phases form the MPA optimization technique are examined. These stages consider varying velocity ratios during the simulation of the complete life of a predator and prey.

The three main optimization phases occur (a) in a high-speed proportion, at which the prey is traveling at a higher pace than the predator; (b) when the prey and predator move at the same pace, forming a velocity ratio of 1; and (c) when the predator is traveling a higher pace than the prey having a minimal ratio of velocity.

Each specified phase is assigned a specific time for iteration. These phases are selected based on the rules governing prey and predator movement while replicating prey and predator natural movement. These three stages are as follows:

Phase 1: At a high-velocity ratio or during the traveling of the predator at a higher pace than the prey. This condition occurs when exploration is vital at the earliest optimization stages. According to the guidelines, the optimum predator approach is to stand still when the velocity ratio between prey and predator is high (v10). The mathematical rule for this model is stated below:

$$Iter<\frac{1}{3}\;Max\_Iter$$

$$\vec{{stepsize}_{i}}=\vec{R_{B}}\times \left(\vec{Elite}-\vec{R_{B}}\times \vec{{Prey}_{i}}\right)\;i=1,\ldots n$$

(6) $$\vec{{Prey}_{i}}=\vec{{Prey}_{i}}+P.\vec{R}\times \vec{{stepsize}_{i}}\;$$

where R_B_ depicts a vector whose values are normal distribution-based and selected randomly, so it depicts Brownian motion. The script defines entry-by-entry multiplications. Prey movement is simulated through the multiplication of R_B_ and prey. P represents a constant with a value of 0.5, and R represents a vector whose values are randomly picked and uniform, ranging from 0 to 1. This occurs in the first third of repetitions in case of high-speed movement or large step size, allowing extensive exploration. Iter represents the present iteration, whereas Max–Iter represents the greatest iterations number.

Phase 2: When the movements of prey and predator have the same pace. It indicates they are searching for prey. This segment occurs during the optimization process’s middle phase, during which exploration tries to mutate transiently to exploitation. Exploitation and exploration are vital at this phase. Therefore, half of the population is designated for exploitation. The other half is earmarked for exploration. At this phase, exploitation is conducted by the prey, while the predator conducts exploration. According to the rule, if the prey travels in a velocity ratio between prey and predator of 1 (v1) in Lévy, Brownian is the ideal predator approach. As a result, in this paper, the predator moves in Brownian, and the prey travels in Lévy.

For the first half of the population: $\frac{1}{3}$ Max_iter < Iter < $\frac{2}{3}$ Max_iter

$$\vec{{stepsize}_{i}}=\vec{R_{L}}\times \left(\vec{Elite}-\vec{R_{B}}\times \vec{{Prey}_{i}}\right)\;i=1,\ldots n/2$$

(7) $$\vec{{Prey}_{i}}=\vec{{Prey}_{i}}+P.\vec{R}\times \vec{{stepsize}_{i}}\;$$

$\vec{R_{L}}$ is a Lévy distribution-based vector of random values that represents Lévy motion. The product of multiplying $\vec{R_{L}}$ and prey replicates prey motion in a Lévy way, whereas adding the prey position and step size simulates prey movement. Since the majority of the Lévy distribution step size is related to tiny strides, exploitation is aided by this part. This analysis anticipates for the other half of the population the following:

$$\vec{{stepsize}_{i}}=\vec{R_{B}}\times \left(\vec{\vec{R_{B}}\times Elite}-\vec{{Prey}_{i}}\right)\;i=n/2,\ldots n$$

(8) $$\vec{{Prey}_{i}}=\vec{{Prey}_{i}}+P.CF\times \vec{{stepsize}_{i}}$$

As $CF={\left(1-\frac{Iter}{Max_{Iter}}\right)}^{\left(2\frac{Iter}{Max_{Iter}}\right)}$ is an adjustable variable used to modify the predator movement’s step size. The products of Elite and R_B_ replicate Brownian motion for predator movement, during which the prey adjusts its location dependent on predator motion in Brownian movement.

Phase 3: The predator is going faster than the prey and at a low-velocity ratio. This case occurs almost at the optimization process’s end, generally linked to strong exploitation ability. For low-velocity ratios (v = 0.1), the Lévy approach is preferred. The phase is described below:

$$Iter<\frac{2}{3}\;Max\_Iter$$

$$\vec{{stepsize}_{i}}=\vec{R_{L}}\times \left(\vec{R_{L}}\times \vec{Elite}-\vec{{Prey}_{i}}\right)\;i=1,\ldots n$$

(9) $$\vec{{Prey}_{i}}=\vec{{Elite}_{i}}+P.CF\times \vec{{stepsize}_{i}}$$

The product of Elite and $\vec{R_{L}}$ mimics predator movement in the Lévy method, while increasing Elite position by step size imitates predator movement, helping the update of the prey’s position.

Based on the rules and principles obtained from various publications, this study replicates the natural movement of prey and predators. These stages mimic the size of steps the predator uses to catch food. According to the guidelines, adopting fixed proportions for Brownian and Lévy movement across the predator’s lifetime is appropriate. The predator movement is still during Phase 1. The motion is Brownian during Phase 2, and the Lévy technique is eventually employed in Phase 3. Similarly, this situation applies to prey since prey, such as silky sharks and tuna fish, can be another possible predator. Both are considered marine predators. However, whereas the silky shark preys on tuna, the tuna feeds on marine invertebrates and bony fish. Prey moves in Phase 1 in Brownian motion, while it moves in Lévy motion during Phase 2. The method that allocates one-third of the iterations to each phase is empirically optimized and yields marginally better outcomes than strategies that alternate between these phases or repeat the stage cyclically.

#### 3.1.3. FAD’s Effect and Eddy Formation

The impact of Fish Aggregating Devices (FADs) and Eddy formation are among the environmental factors that influence changes in the behavior of marine predators. During 20% of their lifetime, sharks will likely take more significant strides in various dimensions to reach an environment with different prey distribution. The other 80% is devoted to the immediate neighborhood of FADs [76]. With the impact of trapping at certain spots in the search space, the FADs are seen as local optima. Local optima stagnation is prevented by considering lengthier steps at the simulation time. As a result, the FAD impact may be expressed numerically as in Equation (10)

(10) $$\vec{{Prey}_{i}}=\left\{\begin{array}{c} \vec{{Prey}_{i}}+CF\left[X_{min}+\vec{R}\times \left(\vec{X_{max}}-\vec{X_{min}}\right)\right]\times \vec{U}\;if\;r\leq FADs\\ \vec{{Prey}_{i}}+\left[FADs+\left(1-r\right)+r\right](\vec{{Prey}_{r1}}-\vec{{Prey}_{r2}})\;if\;r>FADs \end{array}\right.\;$$

The likelihood of FADs influencing the optimization process is depicted by a value of 0.2 for FADs. $\vec{U}$ consists the arrays consisting of zero and one forming a binary vector. This is made by establishing a vector of a random value between 0 and 1, and in case it is less than 0.2, its array is adjusted to zero, and the array is altered to one if it becomes higher than 1. In [0, 1], r is the random uniform number. The vectors $\vec{X_{max}}$ and $\vec{X_{min}}$ contain the higher and lower boundaries of the dimensions. Random prey matrix indexes are represented as subscripts r_1_ and r_2_.

#### 3.1.4. Marine Memory

According to the highlighted elements, marine predators possess an excellent ability to recall where they fed successfully. This memory saving in MPA simulates this competence. This matrix is examined for fitness after adding Fads impact and updating prey, and then Elite is updated. A comparison is made between the fitness of the solution at each current iteration and the previous one, and the answer is replaced by the current one in which it is better suited. This technique enhances the solution quality with each iteration [77] and replicates predators returning after successful foraging to prey-abundant areas. The flowchart of MPA is presented in Figure 6.

### 3.2. Grey Wolf Optimization Algorithm

The ranking behavior of grey wolves, who live in groups of up to 12 wolves, influenced GWO, as documented in [78]. To replicate GWO’s leadership structure, this algorithm provides four levels: alpha, beta, delta, and omega. Alpha refers to a group’s leaders, with the alpha having major responsibility for choices such as hunting and sleeping. Beta is offered to assist alpha in making decisions, with feedback suggestions being its major function. Delta assumes the duties of scouts, carers, elders, and hunters by morphing into alpha and beta wolves. Omega is governed by beta. Every other wolf is required to obey the omega-ranked wolf. The top three ranks direct the hunting procedure in the GWO, and the lower-ranked wolves follow them. GWO’s encircling behavior is calculated using the following formula [78]:

(11) $$\vec{Z}\left(t+1\right)=\vec{Z_{P}}\left(t\right)+\vec{U}.\vec{Y}$$

where $\vec{U}$, $\vec{Y}$ are coefficient vectors, the prey’s position vector is denoted as $\vec{Z_{P}}$. The position of wolves in d-dimensional space is represented by Z, where d is the number of variables, (t) is the number of iterations, and Y is defined by the following equation [78]:

(12) $$\vec{Y}=|\vec{W}.\vec{Z_{P}}\left(t\right)-\bar{Z}\left(t\right)|$$

where $\vec{A}$ and $\vec{C}$ are represented as follows:

(13) $$\vec{U}=2\vec{u}\;.\;\vec{r_{1}}.-\vec{u}$$

(14) $$\vec{W}=2\;.\;\vec{r_{2}}$$

where r_1_, r_2_ are created randomly from the range [0, 1]. The value of $\vec{u}$ decreases consistently between 2 and 0 across iterations. In the grey wolf hunting process, the best contenders for the answer are alpha, beta, and delta, who are shown to be aware of the prey’s likely position. Consequently, the three best solutions identified up to that iteration are kept, causing other wolves to alter their hunting positions to match the ideal area. The method for updating locations is as follows [78]:

(15) $$\vec{Z}\left(t+1\right)=\frac{\vec{z_{1}+z_{2}+z_{3}}}{3}$$

where $z_{1}$, $z_{2}$, $z_{3}$ are calculated using the below equations [78]:

(16) $$\vec{z_{1}}=\vec{Z_{\alpha }}-U_{1}\;.\;(\vec{Y_{\alpha }})$$

(17) $$\vec{z_{2}}=\vec{Z_{\beta }}-U_{2}\;.\;(\vec{Y_{\beta }})$$

(18) $$\vec{z_{3}}=\vec{Z_{\delta }}-U_{3}\;.\;(\vec{Y_{\delta }})$$

where $\vec{z_{1}}$, $\vec{z_{2}}$, $\vec{z_{3}}$ represent the finest solutions in the pack at certain iteration t. Meanwhile, $U_{1}$, $U_{2}$, $U_{3}$ are depicted using Equation (13), while $\vec{Y_{\alpha }}$, $\vec{Y_{\beta }}$, $\vec{Y_{\delta }}$ are depicted using the below equations [79]:

(19) $$\vec{Y_{\alpha }}=|\vec{W_{1}}.\vec{Z_{\alpha }}\left(t\right)-\vec{Z}|$$

(20) $$\vec{Y_{\beta }}=|\vec{W_{2}}.\vec{Z_{\beta }}\left(t\right)-\vec{Z}|$$

(21) $$\vec{Y_{\delta }}=|\vec{W_{3}}.\vec{Z_{\delta }}\left(t\right)-\vec{Z}|$$

where $\vec{W_{1}}$, $\vec{W_{2}}$, $\vec{W_{3}}$ are calculated according to Equation (14).

The vector $\vec{u}$ is a critical component of GWO for regulating exploration and hunting. It is suggested in the original publication of this strategy to minimize $\vec{u}$. To update it, use the following equation [78]:

(22) $$\vec{u}=2-t\;.\frac{2}{\max_{i}ter}$$

where *t* denotes the number of iterations and *ter* is the total number of optimization iterations.

## 4. Results

**Figure 7:**
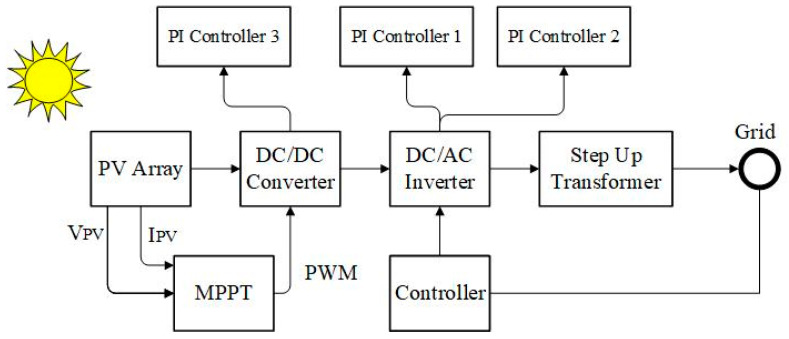
System understudy.

The MPA algorithm is used to figure out the proportional and integral time gains (k_p1_, k_i1_, k_p2_, k_i2_) of the grid-side inverter PI controllers (PI_1_, PI_2_) with k_p1_, k_i1_ being the parameters of the PI_1_ controller responsible for the current regulator, and the voltage regulator is controlled by the PI_2_ controller whose parameters are k_p2_, k_i2,_ with the objective function being obtaining the minimal discrepancy separating the actual and maximum voltage for the system. The control scheme is shown in Figure 7. The higher and lower limits of the variables for the system are shown in Table 4. These boundaries were set based on the survey of former papers and the researcher’s previous research [68]. The system code is presented in Appendix A.

The system has a double transmission line connecting it to the grid, and a 3LG fault is applied to the system at 5 s.

The LVRT features in this study are according to the codes of the German grid [78,79,80,81].

**Figure 8:**
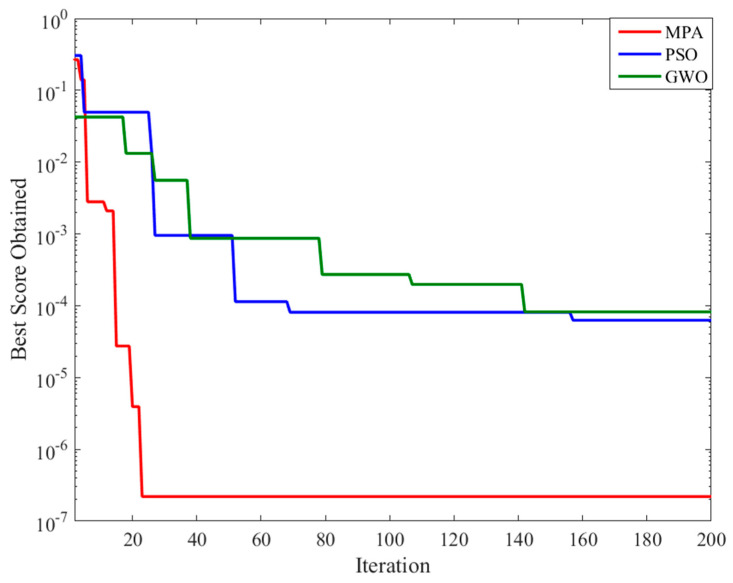
Convergence curves of MPA, PSO, and GWO for the system.

The optimum values for the abovementioned variables are obtained through MATLAB 2020a on a PC with 8 GB RAM and Intel^®^ Core ™ i5 @1.60 GHz processor. The system was run 30 times, and the statistical analysis is provided in Table 5. The optimal value received by the MPA algorithm at the number of iterations equal to 200, shown in Figure 8, was 2.201 × 10^−7^. These optimum values are mentioned in Table 6. The technique parameters for PSO and GWO were obtained as suggested in [82,83]. By repeating the preceding stages, the DC–DC converter PI-3 controller is constructed. The goal is to reduce the difference between the planned system’s actual and maximum voltage. Meanwhile, k_p3_, and k_i3_ are the design input variables.

## 5. Discussion

**Figure 9:**
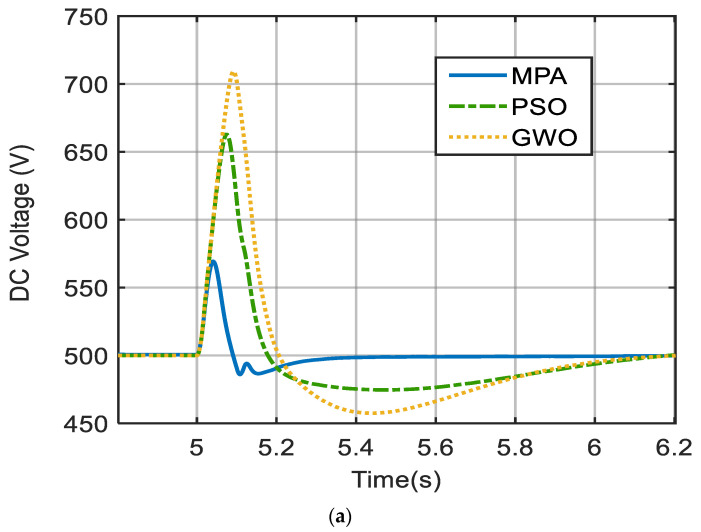

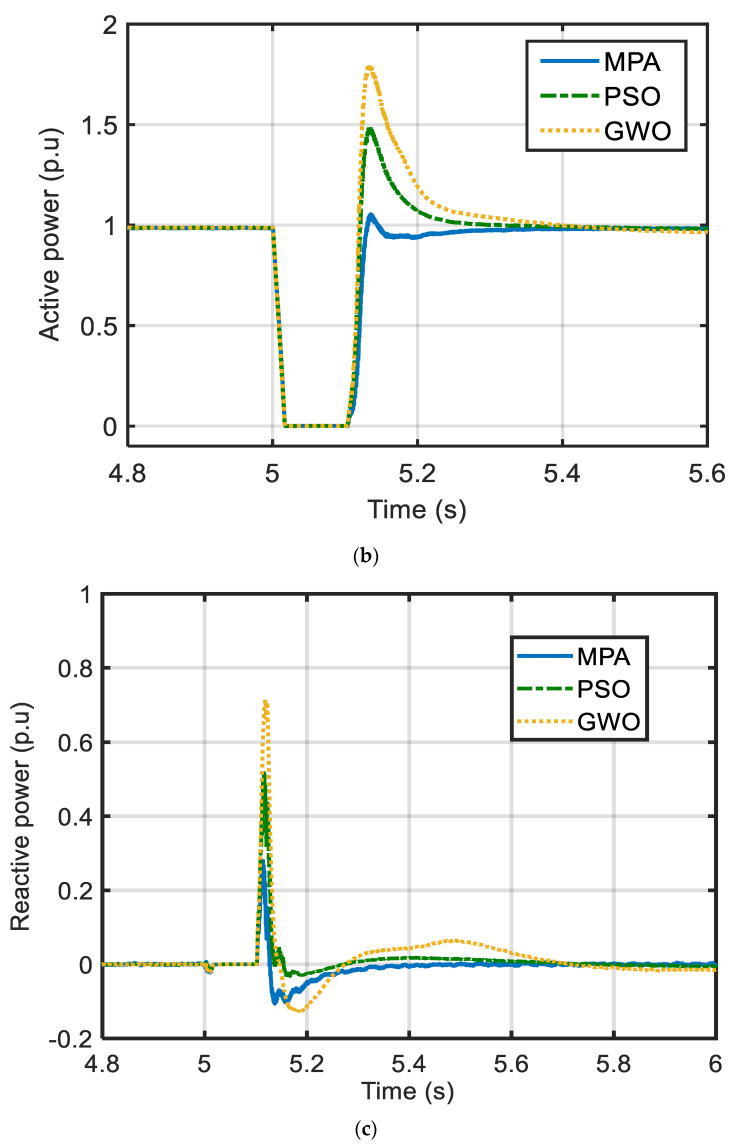

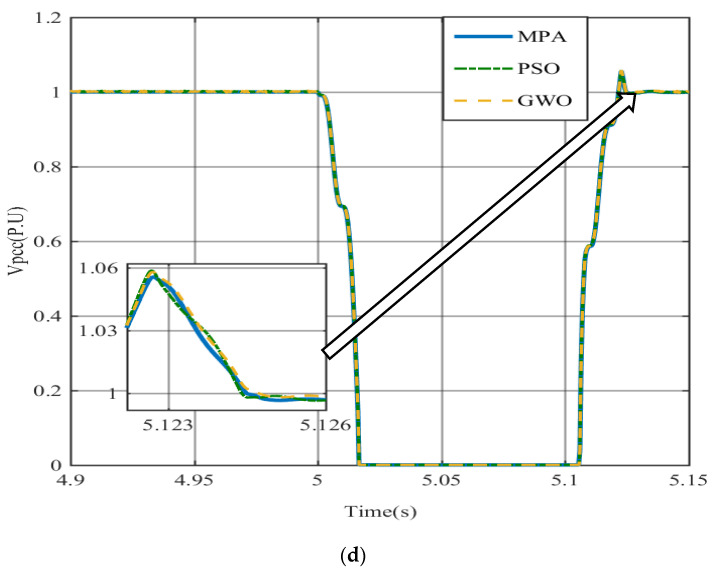
Comparison of responses of PV system under 3L-G symmetrical fault with PSO and GWO. (**a**) V_DC_, (**b**) P at PCC, (**c**) Q at PCC, (**d**) V_PCC_.

The MPA-based PI control technique’s comparative advantage is demonstrated by subjecting a 3LG symmetrical fault on the system and comparing the results to those achieved using two of the most famous optimization techniques, GWO and PSO. The comparison was done in a MATLAB environment. The optimum values using the two techniques are introduced in Table 5. A symmetrical fault at 5.1 s and lasting for 0.9 s is inflicted on the system as indicated in Figure 9b. Installed circuit breakers on the faulty wire are tripped in 0.2 s to remove the fault and reclosed in 1 s.

When the fault occurs, this voltage drops instantly and dramatically. The suggested control mechanism injects reactive power through the grid-side inverter to support the grid. Figure 9a demonstrates the proposed control capacity to keep the voltage of the DC-link fixed with MPA having less overshoot by 14% than PSO and 20% than GWO and also MPA reaching steady state in time less than that of PSO and GWO by 0.8 s. Figure 9b depicts the PCC real power output (P_PCC_) with MPA having less overshoot by 27% than PSO and 40% than GWO and MPA reaching steady state in time less than PSO by 0.76 s and less than GWO by 0.96 s. The reactive power injected at the PCC (Q_PCC_) is shown in Figure 9c, with MPA having overshoot less than PSO by 1.81 times and less than GWO by 2.5 times and reaching a steady state faster than PSO by 0.3 s and quicker than GWO by 0.5 s. Figure 9d depicts the effect of the failure on the V_PCC_, as well as the control reaction to maintaining the V_PCC_. The system was able to sustain stability before 1500 ms, following grid code criteria. The reaction time of the control system is quick and fluctuates very little, reaching a maximum overshoot of less than 1.25 PU in all cases, which is far better than other techniques. The outputs acquired from the proposed MPA-based PI control approach (V_DC_, P_PCC_, Q_PCC,_ and V_PCC_) are compared in the figures mentioned with those obtained from the PSO- and GWO-based PI controllers for a detailed verification of the system response.

**Figure 10:**
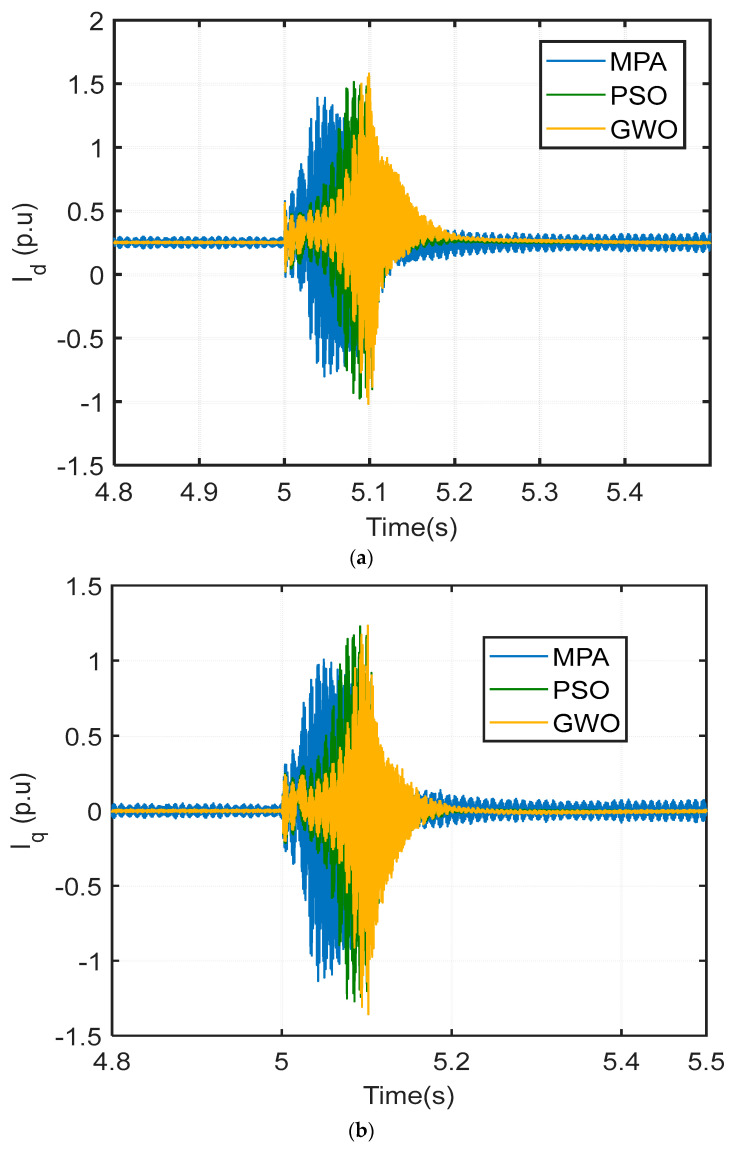
Currents of grid-side inverter under 3LG symmetrical fault conditions.

Further comparisons regarding settling time, overshoot, and steady-state error are shown in Figure 10a,b. The currents of the grid-side inverter, direct axis (I_d_), and quadrature axis (I_q_) utilizing MPA-based, PSO-based, and GWO-based PI controllers are shown in the figures below. The graphs show that the suggested MPA has reduced temporal transient circumstances such as settling time, overshoot, and steady-state error. The currents of the inverter remained within acceptable values during the fault. These previous comparisons show better LVRT capacity and system and dependability for the MPA-based PI control system. The advantage of the suggested control strategy is due to the MPA’s precision in reaching global minima and its good design.

## 6. Conclusions

This work offered a new way to improve LVRT capability using a relatively new MPA-based optimum PI controller. The response of the PCC voltage shown in the MPOS is optimized by modifying the PV system PI controller settings. The system’s responses under the proposed control strategy met all the requirements of the German grid code for enhancing LVRT capabilities when the studied system was exposed to symmetrical fault situations. By comparing the findings with those of the GWO and PSO approaches to validate the suggested strategy, the controller used thoroughly was shown to be quicker, with better damping and fewer fluctuations than GWO and PSO. Finally, the MPA-based PI control technique remarkably improved the LVRT capabilities of PV systems.

Furthermore, the settling time and overshoot of the system in the case of DC-link voltage, active and reactive power, and PCC voltage were found to be very acceptable in the case of the proposed technique. Those results were further verified by comparing them to outcomes from PSO and GWO. The proposed MPA succeeded in keeping the voltage of the DC-link fixed with less overshoot by 14% than PSO and 20% than GWO. Moreover, the proposed MPA method reached a steady state in time less than that of PSO and GWO by 0.8 s. At the same time, the MPA succeeded in enhancing the PCC real power output with an overshoot of less than PSO by 27% and less than GWO by 40%. Also, MPA reached a steady state in time less than PSO by 0.76 s and less than GWO by 0.96 s. The MPA enhanced the reactive power injected, where the overshoot is less than PSO by 1.81 times and less than GWO by 2.5 times. Also, the proposed MPA reached a steady state faster than PSO by 0.3 s and quicker than GWO by 0.5 s. The fitness value was found to be 2.201 × 10^−7^. Therefore, an efficient on-grid PV system was concluded using the control strategy based on the MPA optimization algorithm.

For suggested future work, further sources like wind energy can be added with more faults in addition to the 3LG fault.

## Figures and Tables

**Table 1:** Datasheet of the used PV module (Kyocera KC200GT) at STC.

Manufacturer	Kyocera
Model	KC200GT
Cell Type	Multicrystal
P_m_ (W)	200
V_m_ (V)	26.3
I_m_ (A)	7.61
V_OC_ (V)	32.9
I_SC_ (A)	8.21
Number of series cells	54
K_i_	0.00318 A/°C
K_v_	−0.123 V/°C

**Table 2:** Specifications of 100 kW PV System.

P_m_ (kW)	100
V_m_ (V)	263
I_m_ (A)	380.5
Number of series-connected modules	10
Number of parallel strings	50

**Table 3:** Ratings of excitation circuit.

DC-link voltage	500 V
DC-link capacitor	50,000 µF
Limiting reactor (machine base)	0.2 + j1.0 pu
Power converter’s device	IGBT
Carrier frequency of PWM	1 kHz
Higher DC voltage limit	0.75 kV (150% of rating)
Lower DC voltage limit	0.25 kV (50% of rating)
Protective device short-circuit parameter for overvoltage	Rsh = 0.2 ohm

**Table 4:** Higher and lower boundaries for the system variables.

System Boundaries	Higher Boundaries	Lower Boundaries
k_p1_	9	0.1
k_i1_	9	0.1
k_p2_	9	0.1
k_i2_	9	0.1
k_p3_	9	0.1
k_i3_	9	0.1

**Table 5:** Statistical analysis of MPA.

Factor	Integral Square Error
Minimum	2.201 × 10^−7^
Maximum	2.35738 × 10^−7^
Median	2.24159 × 10^−7^
Average	2.24759 × 10^−7^
Standard Deviation	3.93938 × 10^−10^
Variance	1.55187 × 10^−19^

**Table 6:** Optimal values of the system variables.

Design Variables	MPA	PSO	GWO
k_p1_	0.3042	0.2641	0.2494
k_i1_	7.4268	3.4492	6.7142
k_p2_	6.0533	1.4964	2.4104
k_i2_	6.8165	5.9161	8.1074
k_p3_	1.75	1.5	1.4
k_i3_	0.82	0.7	0.65

## Data Availability

Data are contained within the article.

## Nomenclature

a	Ideality factor of diode
*I_m_*	Maximum output current of PV Array (A)
I_o_	Reverse saturation current of diode (A)
I_ph_	Photo-generated current (A)
*I_sc_*	*Short circuit current of PV module (A)*
k	Boltzmann constant (1.38065e-23 J/K)
K_i_	Short-circuit current coefficient
N_s_	Number of the series connected cells in the module
P_m_	Maximum Output power of PV Module (W)
q	Electron charge (1.6022e-19C)
R_s_	Series resistance (Ω)
R_p_	Shunt resistance (Ω)
T	Cell Temperature (K)
*V_m_*	Maximum output voltage of PV Array (V)
*V_oc_*	Open circuit voltage of PV module (V)
V_th_	Thermal voltage
K_ref_	Reference Duty Cycle
K_M_	Proportionality constant
V_OC-Pilot_	Pilot Module Open circuit voltage
V_O-conv_	DC Converter Output Voltage
I_d-ref_	Reference current of direct axis
I_q-ref_	Reference current of quadrature axis
I_d_	Actual current of direct axis
I_q_	Quadrature current of direct axis
V_PCC_	Voltage of point of common coupling
K_p1_	Proportional Gain of controller 1
K_i1_	Integral Gain of controller 1
K_p2_	Proportional Gain of controller 2
K_i2_	Integral Gain of controller 2
K_p3_	Proportional Gain of controller 3
K_i3_	Integral Gain of controller 3
V_DC_	DC-link Voltage
P_PCC_	Real Power Output at point of common coupling
Q_PCC_	Reactive power injected at point of common coupling

## Appendix A

Used Code

clear all

clc

format long

global Ts

global Ts_Control

global Ts_Power

Ts = 0.00001;

Ts_Control = 0.00001;

Ts_Power = 0.00001;

global Kp1

global Ki1

global Kp2

global Ki2

global Kp3

global Ki3

SearchAgents_no = 30; % Number of search agents

Function_name = ‘F1’;

Max_iteration = 200; % Maximum number of iterations

[lb,ub,dim,fobj] = Get_Functions_details(Function_name);

[Best_score,Best_pos,Convergence_curve] = MPA(SearchAgents_no,Max_iteration,lb,ub,dim,fobj);

function topology

figure(‘Position’,[500 400 700 290])

subplot(1,2,1);

func_plot(F1);

title(‘Function Topology’)

xlabel(‘x_1’);

ylabel(‘x_2’);

zlabel([f1,‘(x_1 , x_2)’])

Convergence curve

subplot(1,2,2);

semilogy(Convergence_curve,‘Color’,‘r’)

title(‘Objective space’)

xlabel(‘Iteration’);

ylabel(‘Best score obtained so far’);

display([‘The best solution obtained by MPA is :’, num2str(Best_pos,10)]);

display([‘The best optimal value of the objective function found by MPA is :’, num2str(Best_score,10)]);

disp(sprintf(‘--------------------------------------’));
